# Beyond knowledge deficits: social visibility, family dynamics, and delayed initiation among people living with HIV in Southwestern China—a descriptive phenomenological study

**DOI:** 10.1186/s12889-026-28278-0

**Published:** 2026-06-30

**Authors:** Ying Zhou, Qilian He, Xin He, Maosong Li, Hongli Yang, Lorna Kwai Ping Suen, Lirong Chen

**Affiliations:** 1 Present Address: School of Nursing, Dali University, Dali, 671000 China; 2 Present Address: Nanchong Central Hospital, Nanchong, 637000 China; 3 Dali Bai Autonomous Prefecture Center for Promoting the Transformation of Scientific and Technological Achievements, Dali, 671013 China; 4 Yunnan Provincial Hospital of Infectious Disease, Kunming, 650000 China; 5 School of Nursing, Tung Wah College, Hong Kong, Hong Kong Special Administrative Region China

**Keywords:** HIV, Antiretroviral therapy, Delayed treatment initiation, Stigma, Qualitative research, Public health, Southwestern China

## Abstract

**Background:**

Despite global recommendations for early antiretroviral therapy (ART) initiation and the well-established benefits of timely treatment, delayed ART initiation remains a concern in some settings. Previous studies have identified multiple barriers to ART initiation, including limited treatment knowledge, stigma, disclosure concerns, side-effect fears, and service-related barriers. Less is known, however, about how these factors become organized in everyday life into a sustained process of delay after diagnosis. This study explored how people living with HIV in southwestern China experienced delayed ART initiation, with attention to social visibility, family relationships, informal information, and changing perceptions of treatment urgency.

**Methods:**

A descriptive phenomenological design was used. Semi-structured, in-depth interviews were conducted with 23 people living with HIV who had delayed ART initiation and were recruited from two HIV-designated hospitals in Yunnan Province, China. Interviews were audio-recorded, transcribed, and analyzed using Colaizzi’s seven-step method. The analysis was primarily inductive, while an HIV-related stigma framework informed the research question, interview domains, and later interpretation.

**Results:**

Four themes were identified. First, delayed ART initiation often began when treatment entry was experienced as a risk of being recognized, leading to concealment, stigma-related shame, and avoidance. Second, delay was sustained through family negotiation, informal information, symptom-free periods, concerns about side effects, and the normalization of waiting. Third, treatment initiation became more likely when family or significant-other support, continued healthcare-provider contact, symptoms, or complications changed participants’ perceptions of risk. Fourth, cross-theme patterns showed that delayed ART initiation was experienced as unfolding from concealment and waiting toward treatment entry when support, symptoms, or changing perceptions of risk made continued delay less acceptable. The local context functioned primarily as a contextual condition shaping social visibility, mobility-related experience, informal information, and treatment urgency, rather than as a standalone mechanism.

**Conclusions:**

Delayed ART initiation in this study was not solely attributable to insufficient knowledge or poor treatment awareness. Participants described delay as an experience shaped by social visibility concerns, stigma-related shame, family negotiation, informal information, symptom experiences, and changing perceptions of risk. Efforts to promote timely ART initiation may need to address not only treatment knowledge, but also confidentiality concerns, stigma-sensitive support, family context, and the social conditions in which treatment decisions are made.

**Supplementary Information:**

The online version contains supplementary material available at 10.1186/s12889-026-28278-0.

## Introduction

Since the World Health Organization (WHO) introduced the “treat all” recommendation in 2015, antiretroviral therapy (ART) has been recommended for all people living with HIV regardless of CD4 cell count [1]. Subsequent WHO guidance has continued to emphasize early and rapid ART initiation as part of public health service delivery, including initiation within seven days of diagnosis and, when appropriate, same-day ART start [2]. Early treatment initiation is associated with improved clinical outcomes and remains a cornerstone of contemporary HIV treatment and prevention strategies [3]. Yet, despite the long-standing global consensus on early treatment and expanding access to HIV services, delayed ART initiation continues to be reported across settings [4]. A recent systematic review and meta-analysis confirmed that delayed ART initiation remains a substantial problem globally, suggesting that policy endorsement of rapid initiation does not automatically translate into timely treatment uptake [5].

Existing studies have identified multiple factors associated with delayed ART initiation, including limited treatment knowledge, concerns about side effects and lifelong medication, financial and logistical barriers, HIV-related stigma, disclosure concerns, and limited social support [6–8]. These studies provide an important basis for understanding why ART initiation may be postponed. However, when these factors are presented mainly as separate barriers, less attention is given to how they become organized in everyday life into a sustained process of delay after diagnosis. In particular, delayed ART initiation may involve not only what barriers are present, but also how individuals interpret treatment entry, manage possible disclosure, negotiate family relationships, and reassess treatment urgency over time. In the context of HIV, treatment decisions are rarely purely biomedical [9–11]. They are often embedded in concerns about identity exposure, social evaluation, moral judgment, family relationships, and the anticipated consequences of being recognized as HIV-positive [12–13]. Recent qualitative evidence from the Treat-All era also suggests that HIV care engagement may increase social visibility, while anticipated, enacted, and internalized stigma can shape whether and how people enter or remain in care. From this perspective, delayed ART initiation can be understood not only as a matter of treatment awareness or service access, but also as a socially situated process of risk appraisal and decision-making [14–16].

These issues may be especially relevant in border settings, where multi-ethnic coexistence, close-knit social networks, population mobility, and cross-regional information flows can shape the social and informational environment in which treatment decisions are made [17–19]. Mobility-related research has shown that movement across places and social environments can affect HIV care engagement, treatment adherence, and continuity of care, highlighting the need to understand how mobility and local context shape treatment trajectories [20–22]. In such settings, treatment decisions may be influenced by concerns about being recognized, informal peer narratives, cross-regional or cross-border experiences, and uncertainty about whether treatment can still be postponed [23–24]. However, in this study, the local border-region context is not treated as an independent causal mechanism. Rather, it is considered part of the broader social and informational environment that may shape social visibility, informal information, family negotiation, and appraisal of treatment urgency [25–27].

Against this background, the present study examines the experiences of delayed ART initiation among people living with HIV in southwestern China. By focusing on how delay was experienced, sustained, and eventually interrupted, the study aims to provide a descriptive, experience-centered account of delayed ART initiation that builds on, rather than replaces, existing evidence on knowledge, stigma, access barriers, and social support.

## Methods

### Study design

This study adopted a descriptive phenomenological design to explore the lived experiences of delayed antiretroviral therapy (ART) initiation among people living with HIV in southwestern China [28]. Descriptive phenomenology was selected because the primary aim was to describe how participants experienced, understood, and responded to delayed ART initiation in everyday life, including diagnosis, concealment, waiting, family negotiation, symptom experiences, and eventual treatment initiation.

Although the study remained grounded in descriptive phenomenology, the findings were organized to describe temporal and relational features that recurred across participants’ accounts. The cross-theme synthesis presented in the Results and Discussion was used to summarize patterns in participants’ lived experiences, rather than to propose a formal theory-generating model or causal pathway.

### Study setting and participants

The study was conducted from January 2025 to October 2025 in two HIV-designated hospitals in Yunnan Province, China. Participants were recruited using purposive sampling guided by the principle of maximum variation. In line with qualitative sampling logic, the study emphasized information richness and experiential variation rather than statistical representativeness.

Eligible participants were those who: (1) had laboratory-confirmed HIV infection [29]; (2) had delayed ART initiation for at least 1 month after diagnosis [30]; (3) had initiated treatment within 1 year after diagnosis; (4) were able to communicate without major barriers; and (5) voluntarily agreed to participate and provided written informed consent. Individuals were excluded if they had a history of severe psychiatric illness, severe physical impairment, or were unwilling to participate.

A total of 26 individuals were recruited. Three individuals were unable to complete the interview process for personal reasons. Finally, 23 participants completed the interviews, and all 23 were included in the final analysis. The final sample covered variation in age, sex, ethnicity, marital status, education, occupation, route of infection, and duration of treatment delay.

The exclusion criterion related to psychiatric illness was applied only to individuals with severe psychiatric conditions that could prevent them from completing an in-depth interview or providing valid informed consent. It was not intended to exclude participants with emotional distress, stigma-related anxiety, or psychological reactions associated with HIV diagnosis and delayed treatment.

### Development of the interview guide

A semi-structured interview guide was developed through literature review and research team discussion. During guide development, the team drew on core dimensions identified in HIV-related stigma research [31], particularly perceived risk of identity exposure, pressure arising from social evaluation, and the influence of stigma-related experiences on delayed treatment initiation. These concepts were used to define the domains of questioning rather than to impose a deductive coding framework. The guide was organized around the experiential process of “diagnosis–delay–turning point–treatment initiation” and remained open-ended to facilitate in-depth narration, consistent with recommendations for phenomenological interviewing. Three pilot interviews were conducted to refine the guide before formal data collection. Core questions addressed why participants did not initiate treatment immediately after diagnosis, what later prompted treatment initiation, how they understood HIV, what emotional and social changes followed diagnosis, whether they received support from others, how disclosure and concealment were negotiated, and how their thoughts changed during the period between diagnosis and treatment initiation. Example questions from the interview guide are provided in Additional file 2.

### Data collection

Data were collected through semi-structured, in-depth interviews conducted in a quiet and private clinic room. All interviewers received training in qualitative interviewing before data collection, including training on HIV-related confidentiality, stigma-sensitive communication, and the use of the interview guide. Before each interview, participants were informed of the purpose of the study and provided written informed consent. With participants’ permission, all interviews were audio-recorded. Interviews were conducted primarily in Mandarin Chinese. Because the inclusion criteria required participants to communicate without major barriers, most participants were able to describe their experiences in Mandarin. When participants occasionally used local dialect terms, place names, kinship terms, border-related expressions, or culturally specific expressions related to stigma, family negotiation, or informal information, local coordinators or research staff familiar with the language and cultural context provided real-time clarification during the interview. These clarifications enabled the interviewers to understand participants’ intended meanings and use follow-up questions appropriately. The clarified meanings were captured in the audio recordings and reflected in the interview transcripts.

Each interview lasted approximately 20–60 min. Follow-up questions were used flexibly according to each participant’s narrative and responses. Interview topics included diagnosis experiences, reasons for delayed treatment, family and social relationships, symptom experiences, and the transition to treatment initiation. During the interviews, the researchers also documented non-verbal expressions, including tone of voice, facial expressions, emotional reactions, and body movements. Audio recordings were transcribed into text within 24 h after each interview. Two members of the research team checked the transcripts for accuracy. During transcript checking, particular attention was paid to locally specific expressions and meanings clarified during the interviews. To protect confidentiality, participants’ names and other identifiable information were replaced with study codes. Data collection and analysis were conducted concurrently. Following each interview, the team carried out rolling coding and comparative analysis to identify newly emerging meaning units and thematic patterns.

For manuscript preparation, selected illustrative quotations were translated from Chinese into English by bilingual members of the research team. The translated quotations were checked against the original Chinese transcripts to ensure semantic accuracy and cultural appropriateness. Full interview transcripts were not translated into English, as data collection, transcription, and analysis were conducted in Chinese.

### Data analysis

Data were analyzed using Colaizzi’s seven-step method [32]. First, all transcripts were read repeatedly to gain an overall understanding of participants’ experiences of delayed ART initiation. Second, significant statements related to diagnosis, concealment, delayed treatment, family interaction, symptom experiences, and treatment initiation were extracted. Third, two researchers independently conducted open coding and organized these significant statements into initial meaning units. Initial codes were generated from participants’ narratives rather than from a predefined coding template.

Fourth, the research team compared initial codes, discussed differences, and refined meaning units through iterative team meetings. Similar meaning units were grouped into subthemes, and subthemes were further organized into broader themes. When coding disagreements occurred, the researchers returned to the original transcripts and field notes, discussed alternative interpretations, and reached consensus. Fifth, the themes were integrated to describe the essential characteristics and internal logic of delayed ART initiation. Sixth, preliminary themes were returned to six participants who were available and willing to provide feedback. These participants generally confirmed that the themes reflected their experiences of delayed treatment and treatment initiation. Their feedback mainly concerned wording clarification and confirmation of locally specific meanings, and no major changes to the thematic structure were required. Seventh, a detailed phenomenological description was produced.

NVivo 15.0 was used to assist data organization and coding. The analysis was primarily inductive. The HIV-related stigma framework informed the formulation of the research question and the development of the interview domains, and it also provided theoretical sensitization for identifying experiences related to identity exposure, social evaluation, internalized stigma, and disclosure concerns. However, it was not used as a rigid deductive coding framework. After the thematic structure had been developed, the research team further examined the temporal and relational features across themes to summarize recurring patterns in participants’ lived experiences. This cross-theme synthesis was treated as a descriptive summary of participants’ narratives and thematic relationships, rather than as a formal theory-generating model or causal pathway.

### Trustworthiness

Several strategies were used to enhance the trustworthiness of the study. First, all audio recordings were transcribed promptly, and transcripts were checked by two members of the research team. During transcript checking, particular attention was paid to locally specific expressions and meanings clarified during the interviews. Second, two researchers independently coded the transcripts, and coding differences were resolved through discussion until consensus was reached. When needed, the researchers returned to the original transcripts and field notes to confirm the interpretation of meaning units and themes.

Third, member checking was used to verify whether the preliminary themes reflected participants’ experiences. Preliminary themes were returned to six participants who were available and willing to provide feedback. These participants generally confirmed that the themes reflected their experiences of delayed treatment and treatment initiation. Their feedback mainly concerned wording clarification and confirmation of locally specific meanings, and no major changes to the thematic structure were required.

Fourth, data collection and analysis were conducted concurrently to support iterative comparison and assessment of information saturation. After approximately the twentieth interview, no substantially new meaning units emerged, and the existing thematic structure was able to account for subsequent interviews. Three additional interviews were conducted to confirm the stability of the themes. Recruitment was stopped after the twenty-third interview when the research team agreed that additional interviews were unlikely to generate new themes. The study was reported with reference to the Consolidated Criteria for Reporting Qualitative Research (COREQ) [33].

### Reflexivity

The research team included investigators with backgrounds in HIV care, nursing, public health, and qualitative research. Interviewers received training in qualitative interviewing, HIV-related confidentiality, and stigma-sensitive communication before data collection. The interviewers were not directly responsible for participants’ clinical treatment, and no prior therapeutic relationship existed between the interviewers and participants. This helped reduce the possibility that participants would feel pressured to provide socially desirable responses because of clinical service relationships.

Before and during data collection and analysis, the research team discussed potential assumptions related to HIV stigma, delayed treatment, family relationships, and the border setting. During analysis, the researchers attempted to bracket these assumptions by prioritizing participants’ own accounts, conducting independent coding, comparing alternative interpretations in team meetings, returning to the original transcripts and field notes when disagreements occurred, and considering less typical or discrepant narratives. These procedures were used to enhance reflexive awareness and reduce the influence of individual assumptions on the interpretation of the data.

## Results

### Participant characteristics

A total of 23 people living with HIV who had delayed antiretroviral therapy (ART) initiation were included in this study. Participants ranged in age from 18 to 75 years and included 16 men and 7 women. They represented diverse ethnic backgrounds, including Han, Bai, Tibetan, Yi, Hui, Miao, Naxi, Dai, Zhuang, and Lisu. Marital status varied across unmarried, married, divorced, and widowed participants. Educational attainment ranged from primary school to undergraduate level, and occupations included unemployed individuals, farmers, employees, temporary workers, retirees, and students. Routes of HIV infection included predominantly heterosexual transmission, as well as homosexual transmission, injection drug use, and blood transmission. The duration of delayed treatment ranged from 2 months to 9 years.

### Qualitative findings

Four major themes were identified from the interview data. Together, these themes showed that delayed ART initiation was not experienced simply as a consequence of insufficient knowledge or a single decision to refuse treatment. Rather, across participants’ narratives, delay began with concerns about social visibility and possible disclosure, was sustained through family negotiation and uncertain treatment urgency, and was later interrupted when support, symptoms, or changing risk perceptions made treatment entry more possible. The fourth theme summarizes recurring patterns across participants’ accounts rather than proposing a formal explanatory model. The themes and subthemes identified from the interview data are summarized in Table 1.

**Table 1:** Summary of themes and subthemes related to delayed ART initiation

Level	Code	Theme / Subtheme	Analytic focus
Theme	**Theme 1**	**Entering concealment: diagnosis as a problem of social visibility**	**Delayed ART initiation began when diagnosis was experienced not only as a medical condition**,** but also as a threat to social identity and everyday relationships. Participants initially tried to manage exposure risk**,** anticipated stigma**,** and the possibility of being recognized as living with HIV.**
Subtheme	1.1	Treatment entry as a risk of being recognized	This subtheme captures how entering ART care, collecting medication, or attending HIV-related services could be perceived as a risk of being seen or identified by others.
Subtheme	1.2	From anticipated stigma to internalized shame	Participants described how anticipated, perceived, or experienced stigma could become internalized as shame, self-blame, emotional withdrawal, or a more negative sense of self.
Subtheme	1.3	Concealment, denial, and avoidance as early self-protective responses	Concealment, denial, and avoidance functioned as early attempts to preserve privacy and social safety after diagnosis, creating the initial conditions for delayed ART initiation.
Theme	**Theme 2**	**Living with delay: family negotiation and uncertain urgency sustained prolonged non-initiation**	**After initial concealment**,** delay was sustained through family negotiation**,** informal information**,** uncertain treatment urgency**,** symptom-free periods**,** and concerns about medication. Waiting became temporarily manageable rather than a fixed refusal of ART.**
Subtheme	2.1	Family relationships as a field of negotiation rather than simple support	Family relationships involved disclosure concerns, emotional burden, shame, responsibility, and care. They could sustain delay while also shaping later treatment decisions.
Subtheme	2.2	Informal information and everyday comparison shaped treatment urgency	Participants interpreted treatment timing through peer experiences, social media, cross-regional or cross-border experience, and everyday comparison, which could reduce the perceived urgency of immediate ART.
Subtheme	2.3	Symptom-free periods and concerns about side effects made waiting feel acceptable	The absence of obvious symptoms, together with fears of medication reactions, made postponement feel acceptable or temporarily justifiable for some participants.
Subtheme	2.4	Waiting became normalized as an everyday condition	Over time, delay became a repeated and familiar way of managing diagnosis, uncertainty, family concerns, informal information, and treatment-related fears.
Theme	**Theme 3**	**Reaching a turning point: when support**,** symptoms**,** or reappraised risk moved patients toward ART**	**Delayed ART initiation was interrupted when family or significant-other support**,** healthcare-provider contact**,** symptoms**,** complications**,** or changing perceptions of risk made treatment entry more possible or necessary.**
Subtheme	3.1	Family and significant-other support helped break the state of waiting	Support, acceptance, or responsibility toward family members helped some participants move beyond waiting and view treatment as necessary for survival and family roles.
Subtheme	3.2	Continued healthcare-provider contact helped reconnect participants to treatment	Sustained and nonjudgmental follow-up from healthcare providers helped reduce isolation, maintain connection to care, and make treatment entry more accessible.
Subtheme	3.3	Symptoms and complications made the risks of delay tangible	Physical symptoms or complications transformed the risks of delayed treatment from abstract possibilities into immediate and embodied concerns.
Subtheme	3.4	Treatment initiation followed a reordering of perceived risk	ART initiation occurred when the perceived risks of continued delay began to outweigh the anticipated social, emotional, or medication-related risks of entering care.
Theme	**Theme 4**	**Patterns across participants’ experiences of delayed ART initiation**	**Summarizes recurring patterns across participants’ accounts without proposing a formal explanatory model or causal pathway.**
Subtheme	4.1	Delay was experienced as unfolding over time	Describes delayed ART initiation as an experience that unfolded from concealment and waiting toward later reconsideration of treatment.
Subtheme	4.2	Social visibility, family relationships, and treatment urgency were interconnected	Shows how social visibility concerns, family relationships, informal information, bodily experience, and treatment urgency were experienced as interconnected.
Subtheme	4.3	The local context appeared mainly as a contextual condition	Clarifies that the local border-region context shaped social and informational conditions but was not treated as a standalone mechanism.
Subtheme	4.4	Delayed ART initiation was not solely attributable to knowledge deficits	Emphasizes that delayed ART initiation was not solely attributable to insufficient knowledge, but reflected social, relational, emotional, and bodily meanings attached to treatment entry.

Note: Bold type indicates theme-level rows; non-bold type indicates subtheme-level rows

#### Theme 1. Entering concealment: diagnosis as a problem of social visibility

Across interviews, many participants described the period immediately after diagnosis as a time when HIV was experienced not only as a medical condition, but also as a threat to social identity and everyday relationships. ART initiation was therefore not always understood as a purely biomedical step. For several participants, entering care raised fears of being recognized, talked about, or judged by others. In this early stage, delayed ART initiation often began as an attempt to manage exposure risk before treatment could be accepted as a necessary part of care.

#### Subtheme 1.1. Treatment entry as a risk of being recognized

Several participants described their initial hesitation to start ART in terms of being seen or identified by others. Their accounts suggested that the act of entering treatment could itself be interpreted as a possible route through which HIV status might become known. In close social environments, participants feared that hospital visits, medication collection, or contact with HIV-related services might make their diagnosis visible.

One participant explained: “I had thought about going to the hospital earlier, but I was afraid that others would know. In a small place like ours, once people know something like this, it spreads very quickly” (P11).

This account illustrates how treatment entry was initially filtered through the possibility of social recognition. The concern was not only about taking medication, but also about what treatment participation might reveal to others. For these participants, delaying treatment functioned as an early attempt to avoid becoming socially visible as a person living with HIV.

#### Subtheme 1.2. From anticipated stigma to internalized shame

For some participants, concerns about being recognized were reinforced by anticipated, perceived, or experienced stigma. Participants described not only fear of gossip or judgment, but also emotional reactions such as shame, self-blame, and withdrawal. In this sense, stigma was not limited to external responses from others; it also shaped how participants understood themselves after diagnosis.

One participant recalled an experience of being treated differently after others became aware of the diagnosis: “When I was hospitalized, the patient in the next bed was friendly on the first day, but after they learned that I had HIV, they started avoiding me. I could feel it” (P17).

This account shows how external reactions and anticipated judgment could be internalized as shame and emotional distance.

#### Subtheme 1.3. Concealment, denial, and avoidance as early self-protective responses

In response to exposure concerns and stigma-related shame, many participants initially tried to protect themselves through concealment, denial, or avoidance. These responses did not necessarily mean that participants rejected ART permanently. Rather, they reflected early efforts to preserve privacy, avoid disclosure, and maintain a sense of social safety after diagnosis.

One participant described the burden of managing the diagnosis alone: “I could never tell other people about this. I did not even dare tell my parents. I had to carry it by myself. What I feared most was that others would find out” (P1).

This type of concealment created the initial conditions for delayed ART initiation. Instead of immediately entering treatment, participants first attempted to contain the social consequences of diagnosis. In this early stage, delay was therefore closely linked to self-protection and avoidance rather than to a simple lack of treatment knowledge.

#### Theme 2. Living with delay: family negotiation and uncertain urgency sustained prolonged non-initiation

After the initial stage of concealment, delayed ART initiation did not simply disappear over time. Across interviews, participants described delay as something that became sustained within everyday life. This prolonged non-initiation was shaped by family negotiation, informal information, uncertain treatment urgency, symptom-free periods, and concerns about medication. In this stage, participants were not necessarily making a fixed decision to refuse ART. Rather, many described a condition in which waiting continued to feel possible, manageable, or temporarily justifiable.

#### Subtheme 2.1. Family relationships as a field of negotiation rather than simple support

Family relationships played a complex role in sustaining delayed ART initiation. For some participants, family was not simply a source of support or non-support. Instead, family life became a relational field in which disclosure, shame, emotional burden, and responsibility had to be negotiated. Several participants delayed treatment because they feared that treatment participation might lead family members to discover their HIV status or become distressed by the diagnosis.

One participant described this concern in relation to parents: “Sometimes I worried that if the hospital called my family because I was taking the medicine here, my parents would find out, and they definitely would not be able to handle it” (P12).

This account shows how family relationships could prolong non-initiation not only through direct opposition, but also through anticipated emotional consequences. For these participants, delaying ART was partly a way to manage family disclosure, protect relatives from distress, and avoid possible disruption in close relationships.

#### Subtheme 2.2. Informal information and everyday comparison shaped treatment urgency

Several participants did not evaluate treatment timing only through formal medical advice. Instead, their sense of urgency was shaped by informal information, peer experiences, social media, and everyday comparison with others who had delayed treatment. These sources could make immediate ART initiation feel less urgent, especially when participants encountered stories of others who had postponed treatment without immediate visible consequences.

One participant explained how peer experience influenced the perception of delay: “When I was using drugs abroad before, I had already come across this kind of medicine. Some people around me also waited several years before starting it, and nothing seemed to happen, so I felt I could delay for a while too” (P2).

For some participants, informal information did not necessarily lead to refusal of treatment. Rather, it shaped how they judged whether treatment needed to begin immediately. In this way, treatment urgency was interpreted through everyday comparison and socially circulated experience, rather than through clinical recommendations alone.

#### Subtheme 2.3. Symptom-free periods and concerns about side effects made waiting feel acceptable

For many participants, the absence of obvious symptoms made delayed ART initiation feel more acceptable. When they did not feel seriously ill, some participants interpreted their condition as still manageable and believed that treatment could be postponed. Concerns about medication side effects further reinforced this sense that waiting was possible.

One participant described how feeling physically well reduced the perceived urgency of treatment: “At the time, my test results were still okay, almost like a normal person’s, so I felt it was not a big problem and decided to leave it for later” (P22). This account illustrates how bodily experience could make waiting feel acceptable. Concerns about medication side effects further reinforced this sense that treatment could still be postponed. Treatment was not rejected outright; rather, it was delayed because the immediate need for ART did not yet feel compelling in participants’ everyday experience.

#### Subtheme 2.4. Waiting became normalized as an everyday condition

Over time, delay could become normalized as part of everyday life. This did not mean that participants had made a permanent decision against treatment. Rather, postponement became a repeated and familiar way of managing diagnosis, uncertainty, and treatment-related concerns. Across interviews, participants described waiting as something that could be extended as long as symptoms remained manageable, disclosure could be avoided, and informal comparisons suggested that immediate treatment might not be necessary.

In this sense, delayed ART initiation became more than an initial act of concealment. It developed into a sustained condition in which non-initiation was repeatedly justified through family concerns, informal information, absence of symptoms, and fear of medication. This normalized waiting formed the context from which later turning points toward treatment initiation emerged.

#### Theme 3. Reaching a turning point: when support, symptoms, or reappraised risk moved patients toward ART

Although delayed ART initiation was sustained for some time, participants’ accounts showed that waiting was not permanent. Across interviews, treatment initiation occurred when the conditions that had supported delay began to change. For some participants, support from family members or significant others reduced the emotional cost of treatment entry. For others, continued contact from healthcare providers helped reconnect them to care. In several accounts, symptoms or complications made the risks of continued delay more immediate. These changes gradually shifted how participants understood the balance between avoiding treatment and entering ART.

#### Subtheme 3.1. Family and significant-other support helped break the state of waiting

For some participants, family relationships that had once been associated with concealment or worry later became an important source of treatment motivation. Support, acceptance, or a renewed sense of responsibility toward family members helped participants move beyond prolonged waiting. In these accounts, treatment initiation became connected not only to personal survival, but also to continuing family roles and responsibilities.

One participant described how responsibility toward an aging parent changed his view of treatment: “I had planned to just die, but in my family there was only my mother and me left. My mother was already over eighty. If I did not stay alive, what would happen to her?” (P3).

This account shows how family responsibility could interrupt the logic of waiting. Rather than simply increasing pressure, family ties could make survival and treatment feel necessary.

#### Subtheme 3.2. Continued healthcare-provider contact helped reconnect participants to treatment

Several participants also described the role of healthcare providers in helping them re-enter care after avoidance or hesitation. Continued contact from clinicians was important not only because it delivered treatment information, but also because it conveyed concern, reduced isolation, and helped participants feel that treatment remained available despite earlier delay.

One participant recalled the effect of repeated follow-up from a doctor: “At first I had decided not to get treated at all. It was Doctor Zhang who kept calling to check on me and persuade me to come for medication. That was when I made up my mind to start treatment” (P20).

This example illustrates how sustained, nonjudgmental contact from healthcare providers could help bridge the gap between avoidance and treatment entry. For participants who had remained outside care, repeated follow-up made the possibility of treatment more concrete and accessible.

#### Subtheme 3.3. Symptoms and complications made the risks of delay tangible

For several participants, physical symptoms or complications became a major turning point. When the body began to feel unwell, the risks of continued delay were no longer abstract or distant. Symptoms made the consequences of non-initiation visible, painful, and difficult to ignore.

One participant described how shingles changed the perceived urgency of treatment: “Before, I really did not want treatment. But when the shingles flared up, it was unbearable, and that was when I truly realized that I could not delay any longer and had to come for treatment” (P9).

This account shows how bodily experience could transform treatment delay from something manageable into something urgent. Symptoms did not simply provide new information; they changed how participants experienced the consequences of waiting.

#### Subtheme 3.4. Treatment initiation followed a reordering of perceived risk

Across participants’ narratives, ART initiation was rarely described as a sudden change of attitude. Rather, it followed a gradual reordering of perceived risk. Earlier in the delay process, the risks of disclosure, stigma, family disruption, or medication side effects could feel more immediate than the risks of HIV progression. Later, as support increased, provider contact continued, or symptoms emerged, continued waiting came to feel less safe.

In this sense, treatment initiation occurred when the perceived costs of delay began to outweigh the anticipated costs of entering care. Participants did not simply move from ignorance to knowledge. Instead, they described a shift in what felt most urgent, most threatening, and most necessary in their everyday lives. This reordering of perceived risk helped explain why ART initiation occurred after a period of concealment and waiting rather than immediately after diagnosis.

#### Theme 4. Patterns across participants’ experiences of delayed ART initiation

Taken together, participants’ accounts showed several recurring patterns across the experience of delayed ART initiation. Delay was not usually described as a single decision to refuse treatment. Rather, participants described an experience that often began with concealment, continued through a period of waiting, and shifted toward treatment entry when support, symptoms, or changing perceptions of risk made continued delay less acceptable. These patterns reflected the interaction of social visibility concerns, family relationships, informal information, bodily experience, and treatment urgency in participants’ everyday lives.

#### Subtheme 4.1. Delay was experienced as unfolding over time

Across interviews, delayed ART initiation was described as unfolding over time rather than occurring as a fixed decision at one point after diagnosis. Participants’ accounts often moved from initial concealment to sustained waiting and later reconsideration of treatment when circumstances changed. This pattern helped explain why treatment entry occurred after a period of delay rather than immediately after diagnosis.

#### Subtheme 4.2. Social visibility, family relationships, and treatment urgency were interconnected

Participants’ accounts suggested that the factors shaping delayed ART initiation were closely connected. Social visibility concerns affected whether treatment entry felt safe; family relationships shaped disclosure, responsibility, and emotional burden; informal information influenced perceptions of treatment timing; and bodily changes altered the meaning of continued waiting. These elements were experienced together rather than as separate barriers.

#### Subtheme 4.3. The local context appeared mainly as a contextual condition

The local context appeared in participants’ narratives mainly as a contextual condition. Its role was reflected in close social networks, population mobility, cross-regional or cross-border life experiences, informal peer narratives, and uncertainty about treatment timing. These contextual features shaped how participants understood exposure, trust, and whether ART could still be postponed. However, the findings do not support treating the border-region context as a standalone mechanism or as evidence of institutional cross-border treatment barriers.

#### Subtheme 4.4. Delayed ART initiation was not solely attributable to knowledge deficits

Overall, participants’ accounts suggested that delayed ART initiation was not solely attributable to insufficient knowledge or poor treatment awareness. Some participants knew that ART was available or had received treatment advice, but still delayed initiation because entering care carried social, relational, emotional, or bodily meanings that had to be managed over time. This interpretation builds on existing evidence by showing how knowledge, stigma, family context, informal information, and changing perceptions of risk were organized in participants’ everyday lives before treatment initiation occurred.

## Discussion

### Delayed ART initiation was not solely a knowledge problem

This study explored how people living with HIV in southwestern China experienced delayed ART initiation. The findings suggest that delayed ART initiation was not solely attributable to insufficient knowledge, poor treatment awareness, or limited service access [34–35]. Instead, participants’ accounts showed that delay was formed and sustained through a sequence of experiences involving social visibility concerns, stigma-related shame, family negotiation, informal information, symptom-free periods, and later changes in perceived risk [36–37].

Previous studies have identified multiple barriers to timely ART initiation, including limited treatment knowledge, concerns about lifelong medication, fear of side effects, stigma, disclosure concerns, and service-related barriers [38–39]. These findings remain important. However, the present study adds to this evidence by showing how such barriers were organized in participants’ everyday lives after diagnosis. Delay was not usually described as a single decision to refuse ART. Rather, it emerged as an experience that began with efforts to preserve privacy, continued through a period of waiting, and shifted toward treatment entry when social, relational, or bodily conditions changed [40–41].

This interpretation helps clarify why delayed ART initiation may persist even when early treatment is recommended and its benefits are known. For some participants, knowing that ART was available did not automatically make treatment entry socially or emotionally manageable. Treatment initiation had to be considered in relation to possible disclosure, family consequences, bodily symptoms, and the perceived urgency of care. In this sense, the findings build on existing evidence about knowledge, stigma, and access barriers by showing how these factors interacted over time in participants’ narratives.

### Social visibility shaped early treatment hesitation

A central finding of this study was that early hesitation to initiate ART was closely linked to concerns about social visibility. Participants often described the period after diagnosis as one in which HIV became a possible source of exposure, recognition, and social judgment. In this context, ART initiation was not experienced only as beginning medication; it could also mean entering designated services, collecting medication, attending follow-up visits, or becoming visible to others as a person living with HIV [42–43].

This finding is consistent with previous research showing that HIV-related stigma, disclosure concerns, and social visibility can influence care engagement and treatment participation [44–45]. In the present study, participants’ accounts suggested that anticipated or experienced stigma could make concealment feel safer than immediate treatment entry, at least during the early period after diagnosis. The concern was not simply whether treatment was understood as beneficial, but whether entering treatment could be managed without unwanted disclosure or social consequences [46]. This does not mean that participants lacked awareness of ART. Rather, their accounts indicated that biomedical urgency competed with perceived social risk. When the possible consequences of being recognized or judged felt immediate, some participants prioritized privacy and concealment before treatment entry [47]. This helps explain why health education alone may be insufficient if confidentiality, stigma-sensitive communication, and disclosure-related concerns are not addressed.

### Family dynamics both sustained delay and supported treatment entry

The findings also highlight the dual role of family relationships in delayed ART initiation. Family was not experienced simply as either a source of support or a source of obstruction. Instead, participants described family as a relational context in which disclosure, shame, emotional burden, responsibility, and care were continually negotiated.

For some participants, family concerns prolonged delay. Treatment participation could raise fears that parents, spouses, or other relatives would discover the diagnosis, become distressed, or experience shame. In these situations, delaying ART was partly a way to manage family disclosure and protect close relationships from disruption [48]. However, family relationships could also become a turning point. Responsibility toward children, aging parents, or spouses, as well as moments of acceptance and encouragement, helped some participants reconsider treatment and move toward ART initiation [49].

This finding adds nuance to previous research that has emphasized social support as a facilitator of HIV care engagement. In this study, family influence was better understood as a negotiated relational process than as a simple supportive or unsupportive factor. Such a perspective may be particularly important for interventions that involve family members. Rather than assuming that family involvement is always beneficial, support strategies may need to consider privacy, readiness for disclosure, emotional burden, and the possibility that family relationships can both sustain waiting and motivate treatment entry [50].

### Local context shaped the interpretation of treatment timing

The local context in this study should be interpreted as a contextual condition rather than as an independent cause of delayed ART initiation. Participants’ accounts did not provide sufficient evidence to support claims about a standalone border-related mechanism or formal institutional barriers to cross-border treatment continuity. Instead, the local border-region context appeared to shape the social and informational environment in which treatment decisions were interpreted.

Several contextual features were relevant. Close social networks could heighten concerns about being recognized. Mobility-related experiences and cross-regional or cross-border life histories exposed some participants to informal peer narratives about ART. Socially circulated information, including stories of others who had delayed treatment without immediate visible consequences, could influence whether treatment seemed urgent. These features did not determine treatment behavior on their own, but they shaped the environment in which participants evaluated exposure, trust, and timing [51].

This interpretation is consistent with mobility-related HIV research suggesting that movement across places and social environments can affect HIV care engagement and treatment trajectories. However, the present study remains cautious in its claims. It does not establish a system-level model of cross-border care discontinuity. Rather, it shows that in this sample, local social and informational conditions contributed to how participants understood delayed ART initiation and the timing of treatment entry.

### ART initiation followed changing perceptions of risk

The findings further suggest that ART initiation occurred when participants’ perceptions of risk changed. Earlier in the delay process, the perceived risks of disclosure, stigma, family disruption, or medication side effects could feel more immediate than the biomedical risks of delaying ART. Later, support from family members or healthcare providers, the emergence of symptoms or complications, and increased awareness of the consequences of waiting made continued delay feel less acceptable [52].

This change should not be understood simply as a movement from ignorance to knowledge. Participants did not always initiate ART because they suddenly learned that treatment was beneficial. Rather, treatment entry became possible when the relative meaning of different risks changed in everyday life. For some, symptoms made the consequences of waiting tangible. For others, family responsibility or healthcare-provider contact reduced the emotional or social cost of entering care [53]. This finding has practical importance. Efforts to promote timely ART initiation may need to help patients not only understand the clinical benefits of ART, but also manage the social and emotional risks they associate with treatment entry. Supporting timely ART initiation may therefore require sustained, nonjudgmental communication, attention to disclosure concerns, reassurance about confidentiality, and support for managing side-effect fears and family-related pressures.

### Practical implications

The findings suggest several implications for efforts to promote timely ART initiation. First, health education remains important, but it may be insufficient if patients continue to perceive treatment entry as a risk of unwanted disclosure or social recognition. Interventions may therefore need to combine treatment education with stronger confidentiality protection, stigma-sensitive communication, and reassurance about privacy during treatment initiation and follow-up.

Second, family-related support should be approached carefully. The findings suggest that family relationships can both sustain delay and support treatment entry. Therefore, family involvement should not be treated as automatically beneficial or universally appropriate. Instead, healthcare providers may need to assess patients’ disclosure readiness, family context, and concerns about emotional burden before involving family members in treatment support. For some patients, protecting privacy may be more important at the early stage, whereas for others, carefully supported family communication may help facilitate treatment initiation.

Third, for patients who remain in a prolonged state of waiting, repeated health education alone may not be enough. Sustained, nonjudgmental contact from healthcare providers may help patients remain connected to care, address fears about side effects, and reconsider the consequences of continued delay. In contexts characterized by mobility and informal information flows, such support may be particularly important for helping patients interpret treatment urgency in relation to both clinical advice and everyday social experience.

### Strengths and limitations

This study has several strengths. It provides an in-depth descriptive phenomenological account of delayed ART initiation among people living with HIV in southwestern China. By using semi-structured interviews and Colaizzi’s analytic procedures, the study describes how delayed treatment was experienced, sustained, and later reconsidered in participants’ everyday lives. The findings also summarize recurring patterns across participants’ accounts, linking social visibility concerns, family negotiation, informal information, bodily experience, and changing perceptions of risk without proposing a formal explanatory model.

Several limitations should be acknowledged. First, participants were recruited from two HIV-designated hospitals, and all had eventually initiated ART. Therefore, the findings may not fully represent people who remain outside treatment systems or who have never initiated ART. Second, although the interviews provided rich accounts of delayed treatment experiences, interview duration varied from approximately 20 to 60 min. Some interviews were relatively short, which may have limited the depth of certain individual narratives. Third, because the study was conducted in a specific setting in southwestern China, transferability to other geographic, cultural, border-region, or health-service contexts should be considered cautiously.

Fourth, the study focused on participants’ retrospective accounts after treatment initiation. Recall bias may have influenced how participants reconstructed their earlier decision-making experiences. Fifth, although the local border-region context was described as part of the broader social and informational environment, the study did not directly examine system-level cross-border care coordination or institutional barriers to treatment continuity. Therefore, the findings should not be interpreted as evidence of a standalone border mechanism. Future research could include people who have not yet initiated ART, compare different geographic settings, or examine mobility and treatment trajectories using longitudinal or multi-site designs.

## Conclusions

This study explored delayed ART initiation among people living with HIV in southwestern China. The findings suggest that delayed ART initiation was not solely attributable to insufficient knowledge or poor treatment awareness. Instead, participants described delay as an experience shaped by concerns about social visibility, stigma-related shame, family negotiation, informal information, symptom experiences, and changing perceptions of risk. Across participants’ accounts, delayed ART initiation was experienced as unfolding from concealment and waiting toward treatment entry when support, symptoms, or changing perceptions of urgency made continued delay less acceptable. Public health efforts to promote timely ART initiation may need to address not only treatment knowledge, but also confidentiality concerns, stigma-sensitive support, family context, and the social conditions in which treatment decisions are made.

## Supplementary Information

Additional File 1. Consolidated criteria for reporting qualitative research (COREQ): 32-item checklist.

Additional File 2. Example questions from the interview guide.

## Data Availability

Relevant excerpts supporting the findings are included in the body of the manuscript. Full interview transcripts are not publicly available because they contain sensitive personal narratives that, even when de-identified, may carry a risk of deductive identification. In addition, permission for public dissemination of raw transcript data was not obtained during the consent process and was not approved by the ethics committee. De-identified data may be available from the corresponding author on reasonable request and subject to approval by the relevant ethics committee.

## Abbreviations

ART: Antiretroviral therapy
COREQ: Consolidated Criteria for Reporting Qualitative Research
HIV: Human immunodeficiency virus
WHO: World Health Organization
