# Supplementary material for: Beyond knowledge deficits: social visibility, family dynamics, and delayed initiation among people living with HIV in Southwestern China—a descriptive phenomenological study
Source: BMC Public Health. 2026 Jun 30;26:2401. doi: 10.1186/s12889-026-28278-0 (PMC13491859; doi:10.1186/s12889-026-28278-0)
Supplement: Supplementary file 2 [file 12889_2026_28278_MOESM2_ESM.docx]

**Example questions from the interview guide**

The following questions illustrate the main domains covered in the semi-structured interviews. The interview guide was used flexibly, and follow-up questions were asked according to participants’ narratives and responses. The questions were designed to explore how delayed ART initiation was experienced, negotiated, prolonged, and eventually interrupted in participants’ everyday lives.

**1. How treatment entry was first experienced: social visibility and exposure risk**

1. Can you describe what happened after you were diagnosed with HIV?

2. What were your first thoughts and feelings when you learned your diagnosis?

3. At that time, how did you understand HIV and its treatment?

4. Did starting treatment feel urgent to you at that time? Why or why not?

5. Did concerns about disclosure, stigma, or being recognized by others affect your thoughts about treatment? If so, how?

6. Did you try to conceal, avoid, deny, or delay dealing with the diagnosis at the beginning? If so, what led you to respond in that way?

**2. How family negotiation, informal information, and uncertain urgency prolonged waiting**

7. Why did you not start antiretroviral therapy immediately after diagnosis?

8. How did your family members or close relationships influence your decision about treatment?

9. Did you receive encouragement, opposition, or mixed messages from family members or other important people?

10. Were there family responsibilities, relational concerns, or other pressures that made treatment more difficult to start?

11. During that period, how did you think about whether treatment needed to be started immediately or could wait?

12. Did information from peers, acquaintances, social media, or other informal sources influence how you understood ART or the timing of treatment? If so, how?

13. Did comparison with other people who delayed treatment affect your own decision?

14. Did your physical condition, absence of symptoms, or concerns about medication side effects affect your decision to wait?

**3. How support, symptoms, and risk reappraisal created turning points toward treatment initiation**

15. During the period between diagnosis and treatment initiation, did your thoughts about treatment change? If so, how?

16. Did any symptoms, health changes, or medical advice make you reconsider treatment?

17. Did support from family members, significant others, peers, or healthcare providers influence your decision?

18. Did continued contact, follow-up, or persuasion from healthcare providers affect your decision to start treatment?

19. Were there particular experiences or events that made you feel treatment had become necessary?

20. What finally prompted you to start treatment?

**4. How delayed treatment initiation unfolded across participants’ experiences**

21. Looking back, how do you now understand the period between diagnosis and treatment initiation?

22. What do you think made treatment delay continue for as long as it did?

23. Did your living environment, local social relationships, mobility experience, or access to information affect how you thought about treatment? If so, how?

24. What kinds of support or conditions might have helped you start treatment earlier?
